# Epigenetics in the Pathogenesis and Treatment of Cutaneous T-Cell Lymphoma

**DOI:** 10.3389/fonc.2021.663961

**Published:** 2021-06-24

**Authors:** Ping Zhang, Mingzhi Zhang

**Affiliations:** ^1^ Department of Oncology, The First Affiliated Hospital of Zhengzhou University, Zhengzhou City, China; ^2^ Department of Oncology, Academy of Medical Sciences of Zhengzhou University, Zhengzhou City, China

**Keywords:** epigenetics, cutaneous T-cell lymphoma, epigenetic biomarkers, HDACi resistance, epigenetic therapy, histone modification, histone deacetylase inhibitor

## Abstract

Cutaneous T-cell lymphomas (CTCLs) comprise a group of heterogeneous diseases involving malignant T cells. The pathogenesis and etiology of CTCL are still unclear, although a large number of genetic and epidemiological studies on CTCL have been conducted. Most CTCLs have an indolent course, making early diagnosis difficult. Once large-cell transformation occurs, CTCL progresses to more aggressive types, resulting in an overall survival of less than five years. Epigenetic drugs, which have shown certain curative effects, have been selected as third-line drugs in patients with relapsing and refractory CTCL. Many studies have also identified epigenetic biomarkers from tissues and peripheral blood of patients with CTCL and suggested that epigenetic changes play a role in malignant transformation and histone deacetylase inhibitor (HDACi) resistance in CTCL. Single-cell sequencing has been applied in CTCL studies, revealing heterogeneity in CTCL malignant T cells. The mechanisms of HDACi resistance have also been described, further facilitating the discovery of novel HDACi targets. Despite the heterogeneity of CTCL disease and its obscure pathogenesis, more epigenetic abnormalities have been gradually discovered recently, which not only enables us to understand CTCL disease further but also improves our understanding of the specific role of epigenetics in the pathogenesis and treatment. In this review, we discuss the recent discoveries concerning the pathological roles of epigenetics and epigenetic therapy in CTCL.

## Introduction

Cutaneous T-cell lymphomas (CTCLs) comprise a heterogeneous group of non-Hodgkin lymphomas derived from skin-homing T cells. Therefore, the classification of CTCL is ill-defined and continuously updated. The incidence of CTCL has been increasing for the past decade and is currently 6.4 per million persons worldwide. The incidence of CTCL increases significantly with age and is thus highest in elderly individuals, especially in patients over 70 years of age (1, 2) Mycosis fungoides (MF) and Sézary syndrome (SS) are the most common types of CTCL, accounting for 50% of CTCLs (3).

Most early stage MF cases have an indolent clinical course. On the other hand, cases of advanced-stage MF (stages IIB–IV) and SS present with an aggressive clinical course, with a median survival of one to five years (4). SS is an aggressive form of CTCL involving mature T cells. It typically has a poor prognosis and limited therapeutic options.

The pathogenesis of CTCL remains elusive. The genetic aberrations and epigenetic modifications associated with the expansion of CTCL T cells have not been elucidated. Recently, next-generation sequencing (NGS) has been used in CTCL studies to offer new insights into the pathogenesis of this condition at the genetic level. Moreover, a large number of studies have shown that the occurrence of CTCL is closely related to epigenetics (5). Epigenetic changes can alter gene expression and function without changing the DNA sequence by regulating gene transcription. These changes mainly involve modification of histones, methylation of DNA, and methylation of microRNA (miRNA) host genes. With the successful clinical application of epigenetic drugs, many investigations have demonstrated the role of epigenetics in CTCL pathogenesis and progression. Emerging data also suggest that treatment strategies aimed at regulating multiple epigenetic targets may be achieved through combination regimens. This review discusses the latest advancements in CTCL, including data on epigenetic markers and their role in CTCL diagnosis, epigenetic therapies for CTCL, and histone deacetylase inhibitor (HDACi) resistance in CTCL.

## Histone Modification in CTCL

**Figure 1:**
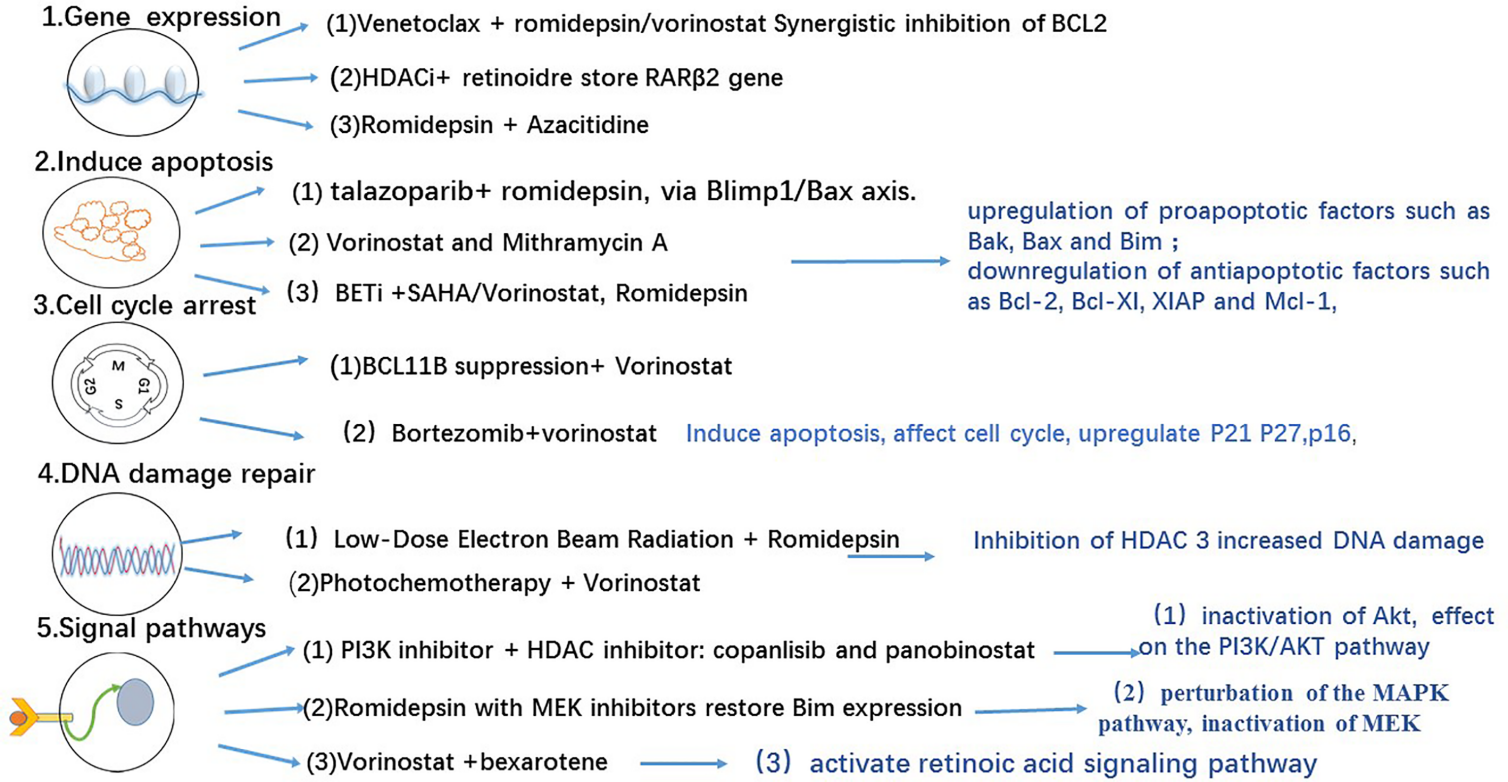
HDACI combination therapy and effects (13).

Gene transcription is regulated by a number of complex enzymes that modify chromatin accessibility. It is controlled by several factors, including the balance between the activities of histone acetyltransferases (HATs) and histone deacetylases (HDACs) that acetylate and deacetylate histones, respectively. HATs facilitate the activation of gene transcription, primarily occurring near the proximal enhancers and promoters. In contrast, HDACs inhibit the expression of specific tumor suppressor genes. Many studies have reported that HDACs consist of 18 subtypes, which can be subdivided into four classes according to their homology with yeast HDACs. Nevertheless, the most frequently studied HDACs in CTCL are HDAC1, HDAC2, and HDAC6 (6). HDAC2 and HDAC6 levels in MF have been reported to be significantly higher than those in healthy individuals. In a study that investigated the differential expression and prognostic significance of HDACs in CTCL, HDAC2 was found to be more highly expressed in the aggressive types of CTCL than in the indolent ones. Meanwhile, HDAC6 overexpression has been associated with favorable outcomes in all CTCL types (7). The overexpression of HDAC1 and HDAC6 may be mediated by IL-15 overexpression in CTCL, making these HDACs attractive molecular targets of IL-15 downstream signaling in CTCL (8). IL-15 is a significant component in the pathogenesis of CTCLs, as the overexpression of IL-15 induces spontaneous CTCL and MF progression (9–11). The inhibition of HDAC6 and the presence of PI3K inhibitors were observed to synergistically inhibit cell proliferation in CTCL cell lines (12) (**Figure 1**). Moreover, HDAC6 inhibition was found to diminish the activation of Akt, a downstream kinase involved in the PI3K pathway (14), and to affect the development of T cells (15). The inhibition of HDAC3 was also reported to be useful in the treatment of CTCL (16). Since HDACs are involved in many signaling pathways that affect cellular death and apoptosis, an increasing number of studies have focused on developing specific HDACis.

### Romidepsin

Romidepsin (depsipeptide) was demonstrated to be effective in patients with CTCL in a study conducted by the National Cancer Institute in 2009 (17). Moreover, two phase II multi-institutional trials showed that romidepsin had a significant single-agent clinical activity against CTCL and yielded robust responses(ORR 34%) in patients with CTCL (18). The NCI1312 clinical trial provided a more comprehensive and detailed description of the responses and tolerated toxicities associated with romidepsin use (**Table 1**). Clinical and correlative data have shown that HDAC inhibitors cannot modify gene expression but affect NF*κ*B target genes. DNA damage may be the dominant mechanism of romidepsin to induce cell death (23). Many retrospective studies have suggested that romidepsin is safe and effective in patients with CTCL who received prior systemic chemotherapy (24, 25). A retrospective comparative analysis of 198 MF/SS patients showed that the median time to next treatment (TTNT) for romidepsin monotherapy (4.5 months) was comparable to that for multi-agent chemotherapy (3.9 months) (26). Long-term use of a dose-reducing romidepsin regimen was also determined to be an alternative treatment strategy in patients with CTCL (27).

**Table 1:** Clinical effects of HDAC Inhibitors in Cutaneous T−cell Lymphoma [refer to Adriana T. Lopez et al. (13)].

Drug	Patients distribution	Response (ORR, DOR, CR)	Response criteria	Approval year
Romidepsin Class I (17)	MF/SS n = 71	ORR = 34% CR = 4 patients (6%) PR = 20 patients (28%) SD=26 patients (38%) PD= 15 patients (17%). Median DOR= 13.7 months TTP = 15.1 months	skin or viscera: RECIST LN: IWG bone marrow, erythroderma, and blood involvement	in 2009, CTCL
Romidepsin (18)	MF; n = 79 SS; n = 17	ORR = 34% CR = 6 patients (6%) PR = 27 patients (28%) SD = 45patients (47%) PD = 10 patients (10%) median DOR = 15 months TTP = 8 months	Skin: SWAT, erythroderma score; LN (RECIST), blood (PFC); pruritus score (100 mmVAS)	
Vorinostat Class I, II (19)	IB–IVA stages MF; n = 44 SS; n = 30	ORR = 29.7% overall; 29.5% in stage IIB or higher; PR = 20 patients CR = 1 patient TTR in stage IIB or higher patients was 56 days Median TTP: 4.9 months overall, 9.8 months for stage IIB or higher responders.	Skin (mSWAT); LN (CT ± PET scan); pruritus score (10-point VAS); blood (PFC)	in 2006, CTCL
Vorinostat (20)	MF; n = 22 SS; n = 11	ORR = 24.2% CR = 0, PR = 8 patients Median DOR = 3.7 months Median TTP = 7.5 months	Skin (mSWAT); LN:(CT ± PET scan); pruritus score (10-point VAS); blood (PFC)	
Belinostat (Class I, II and IV HDACs inhibitor) (21)	MF; n = 17 SS; n = 7	ORR = 13.8% CR = 10.3% Median DOR = 2.72 months Median TTP = 1.41 months	Skin: SWAT LN: International Working Group (IWG)	In 2014, CTCL
Panobinostat Class I, II, IV (22)	MF; n = 105 SS; n = 33 GSSS; n = 1	ORR = 17.3% CR = 1.4% Median DOR = 5.6 months Median PF = 4.2 (bexarotene exposed), 3.7 (bexarotene-naive)	modified Severity Weighted Assessment Tool (mSWAT)	Phase II trial, R/R CTCL

### Vorinostat

Vorinostat, an oral HDACi, was approved by the Food and Drug Administration (FDA) in 2006 for use in the treatment of MF and SS. Many small clinical trials involving the use of vorinostat in patients with CTCL have been performed consecutively (28, 29). The overall response rate of vorinostat in patients with advanced refractory CTCL was 24–30%. According to these trials, vorinostat, which was found to cause reversible side effects, is a safe and well-tolerated third-line drug for patients with CTCL (30, 31). Therefore, many trials that investigated combination therapies with vorinostat have been conducted (32). Some of these combinations have been shown to induce genetic changes, causing malignant cell death, but their effectiveness in the clinical setting is still uncertain (33–35).

### Belinostat

Patients with CTCL who had received one or more prior systemic therapies were enrolled in a belinostat phase II trial (21). The effects of belinostat are listed in **Table 1**.

### HDACi Combination Therapy

The aforementioned epigenetic drugs and their efficacies have been well studied in advanced CTCL. Other effective epigenetic drugs are currently being investigated (36) in patients with advanced MF/SS with variable courses and poor outcomes. Domatinostat, panobinostat, remetinostat, and andresminostat have also been studied in patients with CTCL. Remetinostat gel is the only HDACi that has been investigated for the treatment of topical cutaneous disease in MF. In phase Ib and II trials of remetinostat, patients with limited-stage (IA–IIA) CTCL benefited from remetinostat gel based on the improvements in the composite assessment of index lesions (CAILs) score and modified severity-weighted assessment tool (mSWAT) score, and the clinically significant reductions in pruritus (37). Synergistic HDACi combination therapies have also been investigated to determine their benefits in terms of objective response and durable response time (37). Results from preclinical findings (38) and a phase I clinical trial show that combining vorinostat and bexarotene can provide clinical relief of pruritus (39). Other HDACi combinations have been investigated in preclinical and clinical studies (40) involving the use of interferon-gamma (IFN*γ*) (41, 42), retinoids (43), ultraviolet A (UV-A) phototherapy (44), extracorporeal photopheresis (ECP) (45), PI3K inhibitors (12, 46) the proteasome inhibitor bortezomib (47), and hypomethylated agents such as azacytidine (48) (**Figure 1**). However, these combinations do not show remarkable outcomes. Nevertheless, other HDACi combination chemotherapies and topical skin treatments have shown promising therapeutic potential in patients with CTCL (44, 49). A number of HDACi combination therapies are currently undergoing clinical trials (**Table 2**).

**Table 2:** HDACi combination therapies under investigation are recruiting for relapsed/refractory cutaneous T-cell lymphomas.

Combination trials	Mechanism of action	Phase	ClinicalTrial.gov ID
Romidepsin + Brentuximab vedotin	HDAC inhibitor + antibody-drug conjugate	I	NCT02616965
Romidepsin + lenalidomide	HDAC inhibitor+ Immunomodulatory drugs	II	NCT02232516
Pralatrexate + Romidepsin	HDAC inhibitor + antifolate	I/II	NCT01947140
Romidepsin + Parsaclisib	HDAC inhibitor + PI3K*δ* inhibitor	I	NCT04774068
Romidepsin + Pembrolizumab	HDAC inhibitor + Immunotherapy	I/II	NCT03278782
Romidepsin	Romidepsin maintenance after Allogeneic Stem Cell Transplantation	I	NCT02512497
Romidepsin + 5-Azacitadine	hypomethylation agent + HDAC inhibitor	I/II	NCT01998035
Romidepsin, CC-486 Dexamethasone,Lenalidomide	HDAC inhibitor + Immunomodulatory drugs	I	NCT04447027

### HDACi Therapy Limitations and Strengths

Few studies have directly compared the efficacy and safety profiles of HDACis in patients with MF/SS. A retrospective study compared the TTNT for romidepsin, vorinostat, and panobinostat in patients with MF/SS and reported that there were no significant differences between HDACi therapies, as the overall median TTNT was 5.5 months (50).

Based on the literature, HDACis exhibit similar toxicity profiles. Adverse events include gastrointestinal disturbance, myelosuppression, transient prolongation of QTc interval, nausea, asthenia/fatigue, histone acetylation in peripheral blood mononuclear cells, and infections. Among these events, the most remarkable are the cardiac events, particularly ST-T segment abnormalities and QTc prolongation (51). Differences in the chemical structures of the inhibitors may contribute to the development of these adverse effects.

The National Comprehensive Cancer Network (NCCN) recommends a wide range of therapies for CTCL; however, curative options for CTCL are limited to autologous stem cell transplantations. Among the recommended therapies are those that use vorinostat and romidepsin for systemic therapy. Studies have shown that vorinostat and romidepsin therapies result in unremarkable outcomes compared with other therapies (52). However, data from the outcomes of these therapies were still able to support the use of HDACis as a third-line therapeutic option in advanced CTCL, without increasing morbidity due to toxicity (53). In the phase III MAVORIC trial (n = 372, with 186 patients treated with vorinostat), mogamulizumab was reported to be more effective than vorinostat. For mogamulizumab and vorinostat, the median progression-free survival (PFS) values were 7.7 and 3.1 months, respectively; objective response rates (ORRs) in the MF cohort were 21 and 7.1%, respectively; and ORRs in the SS cohort were 37and 4.1%, respectively (54). A subsequent study compared mogamulizumab and vorinostat in terms of quality of life (QOL) measurements and showed that mogamulizumab was superior to vorinostat. This study also demonstrated that mogamulizumab exhibited a frequency of adverse events that was almost twice as high as that of vorinostat and showed inferior tolerability compared to vorinostat in patients with MF/SS (55). These findings of poor tolerance and adverse effects, such as frequent granulomatous drug eruption, may influence the preference for mogamulizumab (56). The effects of HDACi are non-specific compared to antibody-targeting drugs such as mogamulizumab. These effects on the pathogenesis of CTCL have been reported in many preclinical studies (57). In addition, the mechanisms of HDACi resistance in terms of the heterogeneity of advanced MF/SS have been investigated (58).

### Predictive Biomarkers for Epigenetic Therapy Responses

**Figure 2:**
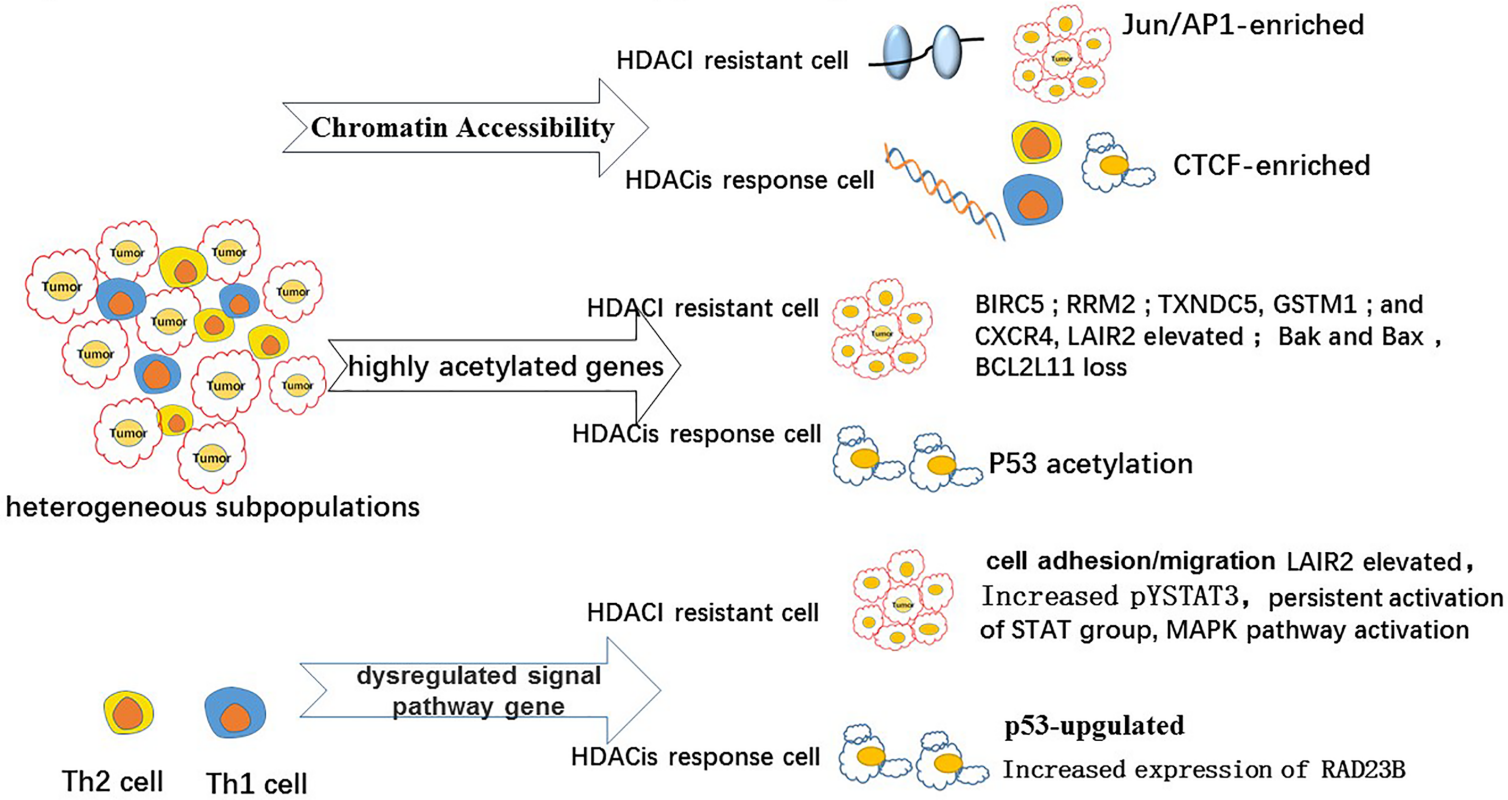
Mechanism of HDACI therapy for drug resistance.

Previous studies have demonstrated that the apoptotic effects of HDACis have a significant role in the treatment of patients with MF/SS. HDACis have been reported to activate intrinsic and extrinsic apoptosis in malignant T cells (59) by increasing the transcription of tumor suppression genes (60), dysregulating cell cycle progression (16), and inhibiting cell proliferation (61). Specifically, a study reported that HDACis induced apoptosis by regulating the expression of pro- and anti-apoptotic genes [p21 (WAF1) and bax] or inducing the transcription of multiple immediate–early (IE) genes (ATF3) (62). Vorinostat affects a wide range of signal pathways (46), including the STAT signaling pathway, and the acetylation of tumor suppressors, including P53 (**Figure 1**) (63). The low overall response rate (approximately 30–40%) of HDACis in CTCL is probably related to HDACi resistance in malignant T cells. Cytogenetic and genomic studies have recently provided data on the molecular mechanism for apoptosis resistance in CTCL malignant T cells and data on the molecular heterogeneity of CTCL cell populations (**Figure 2** **)** (58). In one study, STAT3 and RAD23B genotypes were reported to influence primary HDACi sensitivity in Sézary cells (64). In another study, persistent activation of STAT1 and pSTAT3 was shown to correlate with resistance to vorinostat in patients with CTCL (65). Other studies showed that increased tyrosine phosphorylation of STAT3 (pYSTAT3) expression reduced the response to suberoylanilide hydroxamic acid (SAHA) in Sézary cells, while increased HR23B expression was identified as a determinant of sensitivity to SAHA (66, 67). HR23B expression evaluation in a unique collection of CTCL biopsies taken from a phase II trial of SAHA suggested that HR23B could be an informative biomarker for predicting clinical responses to HDAC inhibitors. These studies also reported that anti-apoptotic cell adhesion/migration genes were highly expressed in MF/SS, suggesting the occurrence of a possible mechanism of HDACi resistance involving the overexpression of such genes in MF/SS. The HDACi resistance genes CCR6, CXCR4, BCL2, BIRC5, CDK1, and LAIR2 were also highly acetylated, possibly inducing the high expression of these genes and promoting disease progression and HDACi resistance. Some of the upregulated genes, STAT4, TNFRSF17, TNFAIP3, GSTM1, GSTM3, and TXNDC5, are known drivers of HDACi resistance (**Figure 2**) (68). In a previous study, LAIR2 was identified as a potential predictor of HDACi resistance in CTCL, as LAIR2 was significantly higher in skin biopsies and blood from patients with HDACi-resistant MF/SS. Mitogen-activated protein kinase (MAPK) pathway activation, which can be reversed by MEK (MAPK kinase) inhibitors, has been speculated to be involved in HDACi resistance (69, 70). Therefore, another probable reason for HDACi resistance is the non-specific off-target effects of HDACis on malignant T cells. Chromatin accessibility has been investigated in CTCL, as changes in chromatin accessibility may predict clinical response to HDACi therapy (69). A study found an association between the clinical response to HDACi treatment and the dynamic increase in DNA accessibility and identified HDACi-responsive (FOXP3) and non-responsive (IFIT3) genes. Both genes were accessible in clinical responders but not in non-responders (shown as **Figure 2**) (71). HDACi resistance may be related to subpopulation heterogeneity within malignant T cells in SS. A high degree of single-cell heterogeneity was identified in SS using single-cell RNA sequencing (72). Terkild Brink et al. demonstrated that distinct subpopulations showed selective sensitivity toward HDACi, causing HDACi resistance (73) that may lead to relapsing or aggressive diseases (73). Although HDACis did not achieve satisfactory responses and had considerable side effects in patients with CTCL (74), the preliminary investigations of HDACi use in CTCL paved the way for novel HDACi drug development for CTCL treatment and provided an increased understanding of the distinct effects and deficiencies of HDACi. In addition, studies on histone modification in CTCL have not only promoted drug discovery and therapy optimization involving HDACis, but also provided a thorough investigation of CTCL epigenomes (75).

## DNA Methylation in CTCL

DNA methylation is one of the most studied epigenetic events in the development and progression of CTCL (76). Methylation abnormalities not only serve as diagnostic biomarkers but also contribute to the development of treatment options for CTCL. DNA methylation is the process catalyzed by DNA methyltransferases (DNMTs), specifically DNMT1, DNMT3A, and DNMT3B, which transfer methyl groups from S-adenosyl methionine (SAM) to the 5-carbon (C5) position of cytosine bases. On the other hand, “epigenetic erasers” from the TET family of proteins are involved in DNA demethylation. Hypermethylation is often associated with gene inactivation and silencing, inhibiting the expression of genes, such as SAMHD1 (77). In CTCL, silenced genes mainly include tumor suppressor genes, including CDKN2A, BCL7A, and MLH1, which regulate cell proliferation and apoptosis resistance during malignant cell transformation (**Table 3**). Defective Fas signaling has been reported as a possible causative agent in MF pathogenesis due to defects in apoptosis signaling in skin-homing T cells. In SS, a reduced Fas expression due to the hypermethylation of the Fas gene promoter region was found to cause apoptosis resistance (89, 94). In contrast, hypomethylation is often associated with genomic instability, leading to gene activation. In a previous study, the demethylation of the SATB1 promoter was found to cause SATB1 overexpression, which promoted malignant T cell proliferation by directly reducing the expression of the inhibitor p21, contributing to the progression of cutaneous CD30+ lymphoproliferative disease (95). Many studies have reported that SATB1 dysregulation is involved in CTCL pathogenesis (96–98). SATB1 is considered a pivotal epigenetic biomarker for CTCL (99), and its dysregulation can be reversed by methyltransferase inhibitors (100). The expression of SATB1 is heterogeneous in CTCL and is associated with the clinical prognosis of patients with MF (101). A reduced SATB1 expression is associated with disease progression and poor prognosis in SS and MF (102). The dysregulation of DNA methylation has been extensively studied in CTCL. Because CTCL presents with a highly variable disease course, CTCL usually cannot be diagnosed before extensive disease progression. To find specific epigenetic markers that can stage and diagnose CTCL, many studies have analyzed the global methylation patterns and epigenetic abnormalities in samples from patients with CTCL, particularly SS (103). The genomic methylation patterns in SS, which are consistent with those of other cancers, are characterized by global genome hypomethylation and specific gene hypermethylation. Hypermethylated promoter CpG loci, such as those in the CMTM2 gene, were found to be sufficient diagnostic biomarkers for SS (92). Specific gene promoter methylation is also a significant predictor of disease diagnosis prognosis, and progression (85) (**Table 3**). Highly expressed PLS3 and TWIST (104, 105) have been well investigated as specific markers for Sézary cells and differentiators between SS and erythrodermic inflammatory dermatoses (EIDs) (106, 107), and these overexpressions have been demonstrated to result from DNA promoter hypomethylation (108). Increased PLS3 was reported to be able to differentiate SS from MF and inflammatory skin diseases and monitor disease progression (107). A broad spectrum of genes, including DNMT3A (109), TET2 (110), CREBBP, MLL, SETDA/B, KDM6B (111, 112), BRD9, SMARCA4, and NuRD (CHD3) (113), involved in epigenetic regulation has been consistently associated with loss-of-function mutations or deletions in CTCL, according to several investigations of whole-genome sequencing (**Table 4**). DNMT3A and TET, which have been frequently reported to be deleted in CTCL, are tumor suppressor genes that play a crucial role in the malignant transformation of mature T cells (114). These genes involved in chromatin modifications were also reported to be highly mutated. ARID1A, which has also been repeatedly shown to be deleted together with ARID5B or SMARC in SS, is a component of the SWI/SNF chromatin remodeling complex and functions as an epigenetic tumor suppressor in CTCL (111, 112). KDM6A, CREBBP, and SETDB2, which are histone-modifying genes, were also reported to be highly mutated in CTCL, based on genomic analysis of 220 cases of CTCLs (115). Both genomic instability and transcriptional dysregulation lead to malignant T-cell transformation and CTCL progression, mostly due to alterations in methylation. Specifically, hypermethylation of the IL-15 promoter region was shown to prevent the binding of the transcriptional repressor Zeb1, increasing the transcription of IL-15 and subsequently initiating CTCL pathogenesis (8).

**Table 3:** Distinct methylation gene of cutaneous T-cell lymphomas and significance [partly refer to Iżykowska K et al. (78)].

Alteration	Markers and Frequency	Significance
Hypomethylation	PLS3 (60% SS, 38% MF) (79), TWIST1 (50%) overexpression	PLS3 overexpression correlates with a better outcome in SS (80) differentiates SS from MF and inflammatory skin diseases
Hypomethylation	GATA6 (28.6%),	GATA6 overexpression induces CD137L overexpression, promoting CTCL cell proliferation, survival, and migration (81)
Hypomethylation	TMEM244 (82)	highest expression in SS
Hypermethylation	p15 (10%), p16 (33%), MGMT (33–36%) (83),	P15, P16 role as tumor suppressors and regulate cell cycle; MGMT encodes DNA repair enzyme (84)
Hypermethylation	PPARG (33%)	PPARG epigenetic silencing can predict early stage MF disease progression (85)
Hypermethylation	BCL7a,PTPRG(27%), P73 (48%), THBS4 (52%) (86)	BCL7a, PTPRG, P73, THBS4 were confirmed as putative tumor suppressor genes in CTCL
Hypermethylation	BCL7a (48%)	BCL7a diminish in MF skin lesions:(1) Significant in separating MF from benign inflammatory skin diseases (87). (2) unfavorable prognostic sign in patients with B-cell lymphoma (88)
Hypermethylation	FAS (89)	Down-regulated FAS reduced sensitivity to apoptosis (90)
Hypermethylation	MLH1 (16%–64%)	silencing of the MLH1 gene induce microsatellite instability, MSI may contribute to disease progression in a subset of tumor stage MF patients (91)
Hypermethylation	CMTM2, C2orf40, G0S2, HSPB6, PROM1, PAM	promoter CpG island hypermethylation of these gene can be diagnostic markers for Sézary syndrome, CMTM2 in particular has 100% sensitivity and specificity (92)
Hypermethylation	RUNX3 (93)	Increased expression of RUNX3/p46 impairs cell viability and induces apoptosis
Hypermethylation	IL15	IL-15 regulates histone deacetylase 1,6 expression, IL-15 roles in cutaneous T-cell lymphoma and promotes progression (8)

**Table 4:** The mutational frequency of epigenetic related gene in CTCL (according to Park J et al. genomic analysis of 220 CTCLs).

Epigenetic Genes	Function	alteration	Frequency
DNMT3A	DNA Methylation	deletion and mutation	4% deletion and 38% mutation
ARID1A,	Nucleosome remodeler	deletion and mutation	5% deletions and 58% mutation
CREBBP	Histone acetylation	mutation	6%
SETD2B	Histone methylation	mutation	28%
TET2	DNA demethylation	mutation	6%
NCOR1	Histone deacetylation	deletion and mutation	3.1 and 80%

## DNA Methylation for Dysregulated miRNAs in CTCL

MiRNAs are a set of small (18–25 bp), single-stranded RNA molecules that regulate gene expression at the post-transcriptional level. With the use of quantitative real-time PCR (RT-qPCR) and comparative genomic hybridization techniques, a differentially expressed miRNA profile has been defined in CTCL (116). Aberrant miRNA levels are common in CTCL. Many investigations have validated miRNA classifiers that can discriminate CTCL from benign inflammation (117) and predict disease progression (118, 119) and prognosis in patients with CTCL (120, 121). Many studies have shown that miRNAs are involved in many signaling pathways that regulate the cell cycle (122) and apoptosis resistance, particularly the Notch, STAT, and (123) NF*κ*B pathogenic pathways. The promoter regions of miR-200c and miR-124-2/3 were reported to be hypermethylated in MF tumor stage (MFt). The repression of miR-200c elevated the expression of Jagged1, contributing to Notch activation in MFt (124). STAT3 is also frequently dysregulated in CTCL (112). STAT3 activation plays a role in CTCL pathogenesis (125) and progression (126) and large-cell transformations (127) MiRNAs, such as miR-337 (123) and miR-124 (128), mediate the expression of STAT3. In particular, miR-124 silencing caused by the hypermethylation of the miR-124 promoter region was shown to increase STAT3 levels in CTCL. Therefore, dysregulated miRNAs are thought to be involved in the pathogenesis of CTCL (129), as miRNA dysregulation is related to miRNA promoter methylation, which has been shown to induce the downregulation of miRNA expression (130). Specifically, DNA methylation in miR-10b, miR-193b, and miR-141 promoter regions was reported to downregulate miRNA expression in CTCL.

## Discussion

Accumulated evidence suggests that intrinsically epigenetic events participate in CTCL malignant transformation and disease progression. Previous reports have identified histone modifications, miRNA regulation, chromatin accessibility aberrations, and abnormal DNA methylation signatures in advanced CTCL, including MF and SS, suggesting that epigenetic dysregulation contributes to the pathogenesis and progression of MF/SS. The expression of epigenetic markers by malignant T cells in CTCL has been extensively exploited to evaluate the role of these markers in diagnosing and managing MF/SS. Stage progression and HDACi resistance have also been discovered to be associated with intratumoral heterogeneity and divergent subclonal evolution. Furthermore, whole‐genome sequencing and single-cell sequencing have been employed to characterize the intratumoral transcriptional heterogeneity of malignant CD4+ T cells, revealing the mechanism of HDACi resistance at the molecular level. Currently, the mechanisms of HDACis are not clear; however, there has been increasing evidence of possible resistance mechanisms. HDACi therapy has already been shown to have positive effects on aggressive CTCL types. HDACis in combination with other therapy, such as chemotherapeutic drugs, immunomodulatory drugs, monoclonal antibody, may provide a novel treatment option that can improve clinical outcomes in patients with CTCL (34, 35, 57, 70, 131–133) (**Table 2**). Currently, more and more HDACI combination therapy regimens are undergoing clinical trials, indicating the important role of HDACi drug in the treatment of CTCL. Indeed, studies on the epigenetic changes in CTCL contribute not only to a comprehensive understanding of CTCL but also to drug development. Moreover, efforts to further elucidate and validate the diagnostic, prognostic, and predictive epigenetic biomarkers for CTCL can facilitate early diagnosis, risk assessment of disease progression, and prediction of treatment outcomes in CTCL. Furthermore, the heterogeneity and diversity of CTCLs allow us to better understand the limitations of HDACi therapy and to optimize HDACi combination therapy.

## Author Contributions

PZ searched and collected papers on Pubmed and wrote the paper. MZ was responsible for reviewing and providing guidance. All authors contributed to the article and approved the submitted version.

## Funding

This work was supported by National Science and Technology Major Project of China (Grant No. 2020ZX09201- 009). This study was supported by National natural Science Foundation of China (81970184, U1904139) and Department of Science & Technology of Henan province (182102310114).

## Conflict of Interest

The authors declare that the research was conducted in the absence of any commercial or financial relationships that could be construed as a potential conflict of interest.
